# Features of Gut Microbiome Associated With Responses to Fecal Microbiota Transplantation for Inflammatory Bowel Disease: A Systematic Review

**DOI:** 10.3389/fmed.2022.773105

**Published:** 2022-05-26

**Authors:** Jindong Zhang, Yangyang Guo, Liping Duan

**Affiliations:** ^1^Department of Gastroenterology, Peking University Third Hospital, Beijing, China; ^2^Department of Critical Care Medicine, Beijing Tiantan Hospital, Capital Medical University, Beijing, China

**Keywords:** gut microbiome, microbial metabolites, fecal microbiota transplantation, response, inflammatory bowel disease

## Abstract

Fecal microbiota transplantation (FMT) has been seen as a novel treatment for inflammatory bowel disease (IBD). The results on microbial alterations and their relationship to treatment efficacy are varied among studies. We performed a systematic review to explore the association between microbial features and therapy outcomes. We searched PubMed, Web of Science, Embase, and Cochrane Library databases from inception to November 2020. Studies that investigated the efficacy of FMT and baseline microbial features or dynamic alteration of the microbiome during FMT were included. The methodological quality of the included cohort studies and randomized controlled trials (RCTs) was assessed using the Newcastle–Ottawa Scale (NOS) and the Cochrane risk of bias tool, respectively. A total of 30 studies were included in the analysis. Compared to non-responders, the microbial structure of patients who responded to FMT had a higher similarity to that of their donors after FMT. Donors of responders (R-d) and non-responders (NR-d) had different microbial taxa, but the results were inconsistent. After FMT, several beneficial short-chain fatty acids- (SCFA-) producing taxa, such as *Faecalibacterium, Eubacterium, Roseburia*, and species belonging to them, were enriched in responders, while pathogenic bacteria (*Escherichia coli* and *Escherichia-Shigella*) belonging to the phylum Proteobacteria were decreased. Alterations of microbial functional genes and metabolites were also observed. In conclusion, the response to FMT was associated with the gut microbiota and their metabolites. The pre-FMT microbial features of recipients, the comparison of pre- and post-FMT microbiota, and the relationship between recipients and donors at baseline should be further investigated using uniform and standardized methods.

## Introduction

Inflammatory bowel disease (IBD) is a chronic and relapsing intestinal disorder that is typically categorized into two subtypes, including ulcerative colitis (UC) and Crohn's disease (CD), and has become a global disease in the 21st century (1). Although the pathophysiological mechanisms of IBD remain unclear, increasing evidence suggests that the disease is caused by the interaction between complex genetic, environmental, and microbial factors, thereby triggering immune-mediated intestinal inflammation (2).

Previous studies have reported the alteration in gut microbiota composition (known as dysbiosis) in patients with IBD, which is characterized by the depletion of *Roseburia hominis, Akkermansia muciniphila, Faecalibacterium prausnitzii*, and *Eubacterium rectale*, and enrichment of *Escherichia coli* (3, 4). Furthermore, patients with IBD exhibit a dramatic alteration in their gut microbiota-derived metabolite profiles compared to the healthy population (5). Based on these findings, therapeutic methods targeting microbiota or their metabolites, such as dietary optimization, probiotics, antibiotics, and fecal microbiota transplantation (FMT), have been applied in clinical practice (6, 7).

Fecal microbiota transplantation has already been recommended to treat recurrent *Clostridium difficile* infection (8). This provides supporting evidence for FMT as a potential treatment method for other intestinal diseases such as IBD. In recent years, there have been increasing studies of the efficacy of FMT for IBD treatment (9), but the clinical outcome is inconsistent among recipients, and the factors affecting its treatment response have been poorly investigated.

With the rapid development of microbiome sequencing technology, more and more researchers have focused on the use of microbiome as a predictive biomarker of clinical outcome and treatment response of FMT (10, 11). Thus, we conducted this systematic review to summarize the current findings on the relationship between microbiota and treatment response of FMT in patients with IBD.

## Materials and Methods

### Search Strategy

A systematic search was conducted in accordance with the Preferred Reporting Items for Systematic Reviews and Meta-Analyses (PRISMA) (12). We searched four databases: PubMed, Web of Science, Embase, and Cochrane Library from inception to 2 November 2020. The search terms covering expressions for fecal, microbiota, transplant, and IBD are listed in the Supplementary Materials.

### Study Selection

Studies were included if they investigated the efficacy of FMT and baseline microbial features or dynamic alteration of the microbiome during FMT in both pediatric and adult patients with IBD.

Studies were excluded if they were: (1) reviews, guidelines, or comments, (2) animal studies, (3) studies that did not involve microbial data, and (4) studies that did not assess treatment response.

### Data Extraction

After excluding studies whose title and abstract clearly did not meet our inclusion criteria, the full text of the remaining studies was reviewed to determine eligibility. The following information was extracted from eligible studies: authors' names, years of publication, country of origin, patient demographics, IBD types and disease activity, donor characteristics, FMT procedure, clinical outcome or treatment response of FMT, and microbial data.

### Quality Assessment

The Newcastle–Ottawa scale (NOS) containing three criteria (selection, comparability, and exposure) was used to assess the quality of the included cohort studies, following the standard 9-point scale, and randomized controlled trials (RCTs) were assessed using the Cochrane risk of bias tool, which incorporate the evaluation of selection, performance, detection, attrition, and reporting bias (13).

## Results

### Study Selection

**Figure 1:**
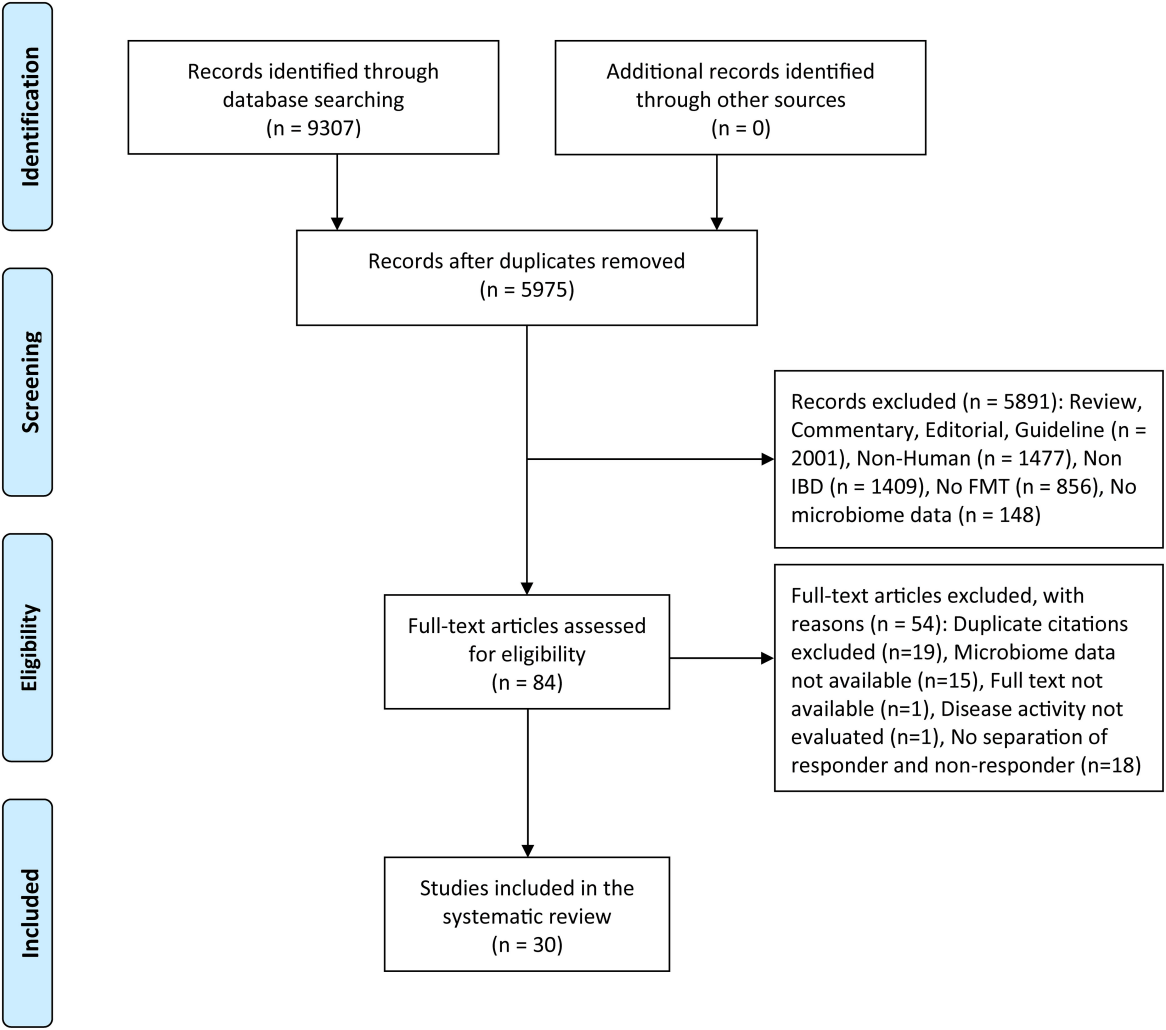
PRISMA flow diagram of the study selection. FMT, fecal microbiota transplantation; IBD, inflammatory Bowel disease.

After initial research, a total of 9,307 records were identified, which were reduced to 5,975 after the removal of internal and external duplicates. Titles and abstracts of 5,975 records were screened, 84 of which were retained for full-text review. Overall, a total of 30 articles or abstracts satisfied the inclusion criteria for this systematic review (Figure 1). The results of the quality assessment for cohort studies and RCTs are presented in Supplementary Tables S1, S2. The quality scores of the 20 studies ranged from 5 to 9 (moderate to high quality). The risk of bias was high in Sokol et al. and Kong et al. because their trials were single-blind, while the remaining studies were at low risk.

### Study Characteristics

The characteristics of the included studies are presented in Supplementary Table S3. Eligible studies included two case reports (14, 15), one case series (16), 20 prospective cohort studies (17, 18), and seven RCTs, of which 29 studies reported on 978 patients, except for one study with no reported patient numbers. A total of 20 studies recruited only patients with UC, six studies recruited only patients with CD, and four studies recruited both the conditions.

### Protocols of FMT

The scope of donor selection and donor stool preparation varied between studies (Table 1). Six studies used pooled donor stool (2–7 donors) to increase microbial diversity while the remaining ones used stool from a single donor. The ratios of stool weight to vehicle volume used for preparation ranged from 1:0.75 to 1:10, and the final volumes of fecal suspension for FMT were 100–500 ml per treatment. Particularly, the studies by Li et al. (11) and Zhang et al. (35) used washed microbiota transplantation (41). Antibiotic pretreatment was used in six studies (22, 26). The colonoscope was the most adopted route by researchers, and the infusion sites included the cecum, terminal ileum, and colon. The frequency of FMT varied between studies.

**Table 1:** Summary of stool preparation and delivery methods.

**References**	**Donor relationship**	**Donor stool**	**Fresh/frozen**	**Stool preparation**	**Dosage per treatment**	**Pre-antibiotics**	**Pre-medication**	**FMT route**	**Region of infusion**	**Number of infusions**
Kao et al. (14)	Unrelated	Single donor	Fresh	1:4 of stool: saline	400 ml	None	None	Colonoscopy	Cecum	1
Shimizu et al. (15)	Related (father)	Single donor	Fresh	Stool diluted in 250 ml saline	250–300 ml	None	None	Colonoscopy for the first time, then enema	Throughout the colon (colonoscopy)	16 (daily for first 5 days, then every 2–4 weeks over 10 months)
Quagliariello et al. (16)	Related (father)	Single donor	Fresh	Stool diluted in saline at ratio 50 g/200 ml	NR	None	None	Colonoscopy	Cecum or duodenum-jejunum	1
Angelberger et al. (17)	Unrelated	Single donor^*^	Fresh	60 g mixed with 250 ml saline	Median: Nasojejunal infusion 24 g; Enema: 20 g	Metronidazole 500 mg bid for 5–10 days	Probiotics^$^, pantoprazole	Nasojejunal tube and enema	Jejunum	3 (daily for 3 consecutive days)
Suskind et al. (18)	Related (parent)	Single donor	Fresh	30 g mixed with 100–200 ml saline	30 g	Rifaximin 200 mg tid for 3 days	Omeprazole	Nasogastric tube	Stomach	1
Vaughn et al. (19)	Unrelated	Single donor	Frozen	50 g mixed with 250 ml saline	250 ml	None	None	Colonoscopy	Terminal ileum to colon	1
Vermeire et al. (20)	Related (sibling or parent), unrelated (friend)	Single donor	Fresh	200 g homogenized with 400 ml saline	400 ml	None	None	Nasojejunal tube or rectal tube	Jejunum; rectum	2
Jacob et al. (21)	Unrelated	Pooled (2 donors)	Frozen	60 ml from each donor pooled	120 ml	None	None	Colonoscopy	Ileum and right colon	1
Ishikawa et al. (22)	Related (spouses or relatives)	Single donor	Fresh	150–250 g diluted with 350–500 ml saline	350–500 ml	Amoxicillin (1,500 mg/d), fosfomycin (3,000 mg/d), metronidazole (750 mg/d) for 2 weeks	None	Colonoscopy	Cecum and ascending colon (2/3 of the volume), transverse colon (1/3 of the volume)	1
Nishida et al. (23)	Related (relatives within the second degree of relationship)	Single donor	Fresh	150–200 g dissolved in 500 ml saline	500 ml	None	None	Colonoscopy	Cecum	1
Goyal et al. (24)	Family members, first-degree relatives, or trusted friends	Single donor	Fresh	150 g stool blended using 250–300 ml saline	Duodenum or jejunum: 20–30 ml; ileum and colon: 200–250 ml	Metronidazole or vancomycin 10 mg/kg tid for 5 days	Omeprazole, loperamide	Colonoscopy	Distal duodenum or proximal jejunum; ileum and right colon	1
Karakan et al. (25)	NR	NR	NR	NR	NR	NR	NR	NR	NR	NR
Kump et al. (26)	Related or unrelated	Single donor	Fresh	50 g stool diluted with 200–500 ml saline	250–500 ml	Vancomycin 250 mg qid, paromomycin 250 mg tid, nystatin 10 ml qid for 10 days	None	Colonoscopy for the first time, then sigmoidoscopy	Terminal ileum and right colon (colonoscopy); left colon (sigmoidoscopy)	5 (in 14 days intervals)
Nusbaum et al. (27)	Family members or friends	Single donor	Fresh	Stool blended in saline	240 ml maximum	None	None	Retention enema	NR	5 (daily for 5 days)
Cold et al. (28)	Unrelated	Pooled (4 donor)	Frozen	Stool homogenized with 500 ml saline, then concentrated and encapsulated	~12 g	None	None	Capsules	Oral administration	25 capsules daily for 50 days
Fan et al. (29)	Unrelated	Pooled (2–3 donor)	NR	NR	NR	NR	NR	Capsules	Oral administration	3 days per week
Gogokhia et al. (30)	Unrelated	Pooled (2 donors)	Frozen	60 ml from each donor pooled	120 ml	None	None	Colonoscopy	Ileum and right colon	1
Gutin et al. (31)	Unrelated	NR	Frozen	NR	250 ml	Rifaximin 550 mg tid for 5 days^#^	None	Colonoscopy	Terminal ileum or neoterminal ileum	1
Chen et al. (32)	Unrelated	Single donor	Frozen	150–200 g stool dissolved in 1,000 ml saline	150 ml (~50 cm^3^ microbiota)	None	None	Transendoscopic enteral tubing (TET)	Entire colon	3
Li et al. (11)	Relatives or friends or unrelated	Single donor	Fresh or frozen	Preparation by automatic microbiota purification system	NR	None	None	Gastroscopy, colonic TET, mid-gut TET	Stomach, Ileocecum, distal duodenum	1
Ohmiya et al. (33)	NR	NR	Fresh	NR	NR	NR	NR	Colonoscopy (UC); oral enteroscopy (CD)	NR	1
Schierová et al. (34)	Unrelated	Single donor	Frozen	50 g dissolved in 150 ml saline	150 ml	None	None	Enema	NR	10 (5 times in the first week, then once a week for 5 weeks)
Zhang et al. (35)	Unrelated	NR	Fresh or frozen	Preparation by automatic microbiota purification system	NR	None	None	NR	NR	NR
Rossen et al. (36) and Fuentes et al. (37)	Partners, relatives, or volunteers	Single donor	Fresh	Median 120 g stool diluted in 500 ml saline	500 ml	None	None	Nasoduodenal tube	Duodenum	2 times at a 3-week interval
Costello et al. (38)	Unrelated	Pooled (3–4 donors)	Frozen	pooled stool (25%) blended with saline (65%) and glycerol (10%)	Colonoscopic delivery: 50 g/200 ml; enema: 25 g/100 ml	None	Loperamide	Coloscopy for the first time, then enema	Right colon (colonoscopy)	3 (coloscopy for the first time, then enema 2 times in the following 7 days)
Sokol et al. (39) and Kong et al. (40)	Unrelated	Single donor	Fresh	50–100 g stool resuspended in 250–350 ml saline	300 ml	None	None	Colonoscopy	NR	1

*CD, Crohn's disease; NR: not recorded; UC, ulcerative colitis*.

* *One patient in this study received fecal microbiota transplantation (FMT) from two different donors*.

# *Three patients in this study used rifaximin while others did not*.

$ *Saccharomyces boulardii or Omnibiotic 6*.

### Clinical Outcomes

Clinical outcomes, including clinical response, clinical remission, and endoscopic remission, are shown in Supplementary Table S4. Follow-up after FMT varied between 1 and 35 months, and the most commonly used endpoint was 12 weeks. In cohort and RCT studies, 18 studies reported the clinical response rate of patients with UC ranging from 20 to 100%, and the clinical response rate of patients with CD reported in seven studies varied between 20 and 75%. The clinical remission rate of patients with UC and CD ranged from 0 to 71.4% and from 10 to 87.5%, respectively. Eight studies reported the endoscopic remission of patients with UC, ranging from 0 to 50%, while only one study on CD reported that no patients achieved endoscopic remission.

### Microbial Sequencing Results

Differences in sample collection and sequencing are listed in Supplementary Table S5. Two studies used both stool and mucosal biopsy specimen for sequencing, and the remaining studies used stool samples. 16S rRNA sequencing was the most adopted method, and other methods included polymerase chain reaction (PCR) and terminal restriction fragment-length polymorphism (T-RFLP) analysis, HITChip, and metagenomic shotgun sequencing. In the case of 16S rRNA sequencing, the 16S rRNA variable regions used for DNA amplification, the sequencing and data analysis platform, and the reference database varied between studies.

#### Microbial Difference Between FMT Donors of Responders and Non-responders

A total of 15 studies reported the relationship between donor gut microbiota and the clinical response (Table 2). Microbial structural similarities between pre-FMT recipients and their donors were lower in responders than in non-responders by Goyal et al. (24) and Cold et al. (28). For post-FMT samples, six studies (19, 24, 27, 29, 34, 36) reported a significant increase in similarity to corresponding donors in responders compared to non-responders, and a case report by Kao et al. (14) also showed that the fecal microbial composition of the patient and the donor closely resembled each other after FMT. Furthermore, Cold et al. (28) found that the microbial composition of responders became closer to their donor than the nonresponders did.

**Table 2:** Association between donor microbiota and the response to FMT.

**References**	**Microbial similarity between pre-FMT patients and donors**	**Microbial similarity between post-FMT patients and donors**	**Comparison between R-d and NR-d**
Kao et al. (14)		↑	
Suskind et al. (18)		Not sure	
Vaughn et al. (19)		R > NR	
Vermeire et al. (20)			Richness: R-d > NR-d
Jacob et al. (21)			Significant difference of structure between R-d and NR-d
Nishida et al. (23)			*Bifidobacterium*: R-d > NR-d; *Lactobacillales, Clostridium cluster IV*, and *Clostridium cluster XI*: R-d < NR-d
Goyal et al. (24)	R < NR (difference not significant)	R > NR	α-diversity: R-d = NR-d; β- diversity: R-d = NR-d
Karakan et al. (25)			Richness: R-d > NR-d; *Akkermansia muciniphila, Faecalibacterium prausnitzii, Ruminococcus*: R-d > NR-d
Kump et al. (26)			Richness and diversity: RE-d > NR-d; significant difference of structure between RE-d and NR-d; Actinobacteria, unclassified *Ruminococcaceae*, an unclassified *Ruminococcus* and *Akkermansia muciniphila*: RE-d > NR-d
Nusbaum et al. (27)		R > NR	
Cold et al. (28)	R < NR	ΔR > ΔNR	
Fan et al. (29)		R > NR	
Schierová et al. (34)		R > NR	
Rossen et al. (36)		R > NR	

*FMT, fecal microbiota transplantation; R, responders; NR, non-responders; R-d, donors of responders; NR-d, donors of non-responders; RE, remission*.

Δ, *alteration degree*.

Several studies compared the microbiota between the donors of responders (R-d) and non-responders (NR-d). Three studies (20, 25, 26) reported higher richness in R-d than in NR-d, while the study by Goyal et al. (24) showed no significant difference in richness between R-d and NR-d. The microbial structure of R-d and NR-d was significantly different in the studies by Jacob et al. (21) and Kump et al. (26), but not in the study by Goyal et al. (24).

In terms of microbial taxa difference, the abundance of *A. muciniphila* and *Runimococcuus*. spp. was elevated in R-d compared to NR-d in two studies consistently (25, 26), and other enriched bacteria phyla or genera included Actinobacteria, unclassified *Ruminococcaceae* (26), *Bifidobacterium* (23), *F. prausnitzii* (25), *Bacteroides fragilis*, and *Bacteroides finegoldii* (10). In addition, the relative abundance of *Lactobacillales, Clostridium cluster IV*, C*lostridium cluster XI* (23), and *Clostridium XIVa* (10) were higher in the feces of the donors of non-responders than that of the donors of responders. Particularly, one study reported that terpenoid backbone biosynthesis pathways in the microbiota were enriched in R-d (10).

#### Microbial Difference Between FMT Responders and Non-responders

##### α-Diversity

The majority of the included studies compared gut microbial diversity and composition between FMT responders and non-responders, by assessing α-diversity and bacterial abundance. Details of these findings are listed in Table 3. As for the α-diversity of pre-FMT samples, the results were discrepant in three studies, presenting higher diversity expressed by the number of OTUs and Shannon index (10), lower diversity reflected by observed OTUs (24) (difference not significant), or no difference (34) in the responders. In three of the seven studies comparing the α-diversity of post-FMT in responders to non-responders, the increasing degree in diversity was significantly greater for responders vs. non-responders (19, 24, 27), two studies showed increased values of α-diversity for responders than for non-responders (10, 36), and only one study reported no difference between responders and non-responders (23).

**Table 3:** Comparison of α-diversity between responders and non-responders.

**References**	**Pre-FMT**	**Post-FMT**	**α-diversity**
			**index**
Vaughn et al. (19)		ΔR > ΔNR	Shannon
Nishida et al. (23)		R = NR; R-d = NR-d	Shannon
Goyal et al. (24)	R < NR (difference not significant)	ΔR > ΔNR	Observed OTUs
Nusbaum et al. (27)		ΔR > ΔNR	Species richness, Shannon, Inverse Simpson
Rossen et al. (36)		R ↑; NR no change	Shannon
Paramsothy et al. (10)	R > NR	R > NR	Number of OTUs, Shannon

*FMT, fecal microbiota transplantation; R, responders; NR, non-responders*.

Δ, *alteration degree*.

##### Baseline Microbiome Composition

Two of the included studies analyzed the association between response and baseline microbiome composition. The study performed by Goyal et al. (24) demonstrated that responders contained a higher relative abundance of *Fusobacterium* than non-responders at baseline, and Gutin et al. (31) observed that the baseline microbiome of responders had higher counts of *Enterobacteriaceae* and *Bifidobacterium* members, whereas non-responders had greater abundance of *Lachnospiraceae* and *Ruminococcaceae* members.

##### Differences in Microbiome Composition Between Responders and Non-responders

A number of differences were observed between responders and non-responders after FMT (Table 4). Several bacteria showed a relatively consistent trend in separate studies, in which the increased microorganisms included the phyla Bacteroidetes (22, 36), the family *Lachnospiraceae* (14, 27, 30, 31, 34), and the genera *Collinsella* (33, 34), *Bacteroides* (14, 15), *Blautia* (14, 34), *Faecalibacterium* (14, 15, 33, 34), *Eubacterium* (11, 15), *Clostridium clusters IV* (36, 42), *Roseburia* (14, 20, 27), and *Ruminococcus* (11, 30, 42). In contrast, the relative abundance of the genera *Enterococcu*s (14, 37), *Lactobacillus* (14, 34), *Veillonella* (10, 37), and *Sutterella* (14, 42) was reported to decrease in responders. For the species level, responders had an increased abundance of the species *Ruminococcus bromii* (10, 16), *Eubacterium hallii* (10, 37), *Eubacterium ventriosum* (19, 37), and *F. prausnitzii* (17, 27, 32), and reduced abundance of species *Bacteroides vulgatus* (19, 37)*, E. coli* (18, 30, 37)*, Escherichia-Shigella* (29, 30), and *Sutterella wadsworthensis* (10, 37). A few of bacteria showed an opposite changing trend in their abundance, including the family *Ruminococcaceae* (33, 34) and *Christensenellaceae* (30, 34), the genus *Escherichia* (10, 15), and the species *Bacteroides ovatus* (16, 17, 19, 37) and *Ruminococcus gnavus* (16, 37).

**Table 4:** Microbial difference between responders and non-responders after FMT.

	**Studies**																					**Total**	
**Microbial taxa**	**14**	**15**	**16**	**17**	**18**	**19**	**20**	**22**	**26**	**27**	**29**	**30**	**31**	**32**	**11**	**33**	**34**	**36**	**37**	**42**	**10**	**↑**	**↓**
**Actinobacteria**																							
*Collinsella*																↑(CD)	↑					2	0
**Bacteroidetes**								↑										↑				2	0
*Bacteroides*	↑	↑																				2	0
*Bacteroides ovatus*			↑	↑		↓													↓			2	2
*Bacteroides vulgatus*						↓													↓			0	2
**Firmicutes**																							
Lachnospiraceae	↑									↑		↑	↑			↓(UC)	↑					5	1
Ruminococcaceae																↓(UC)	↑					1	1
Christensenellaceae												↑					↓					1	1
*Blautia*	↑																↑					2	0
*Faecalibacterium*	↑	↑														↑(UC)	↑					4	0
*Eubacterium*		↑													↑							2	0
*Clostridium clusters IV*																		↑		↑		2	0
*Clostridium clusters XIVa*																		↑	↑		↓	2	1
*Roseburia*	↑						↑(UC)			↑												3	0
*Enterococcus*	↓																		↓			0	2
*Lactobacillus*	↓																↓					0	2
*Ruminococcus*												↑			↑					↑		3	0
*Veillonella*																			↓		↓	0	2
*Dialister*							↑(CD)		↓												↓	1	2
*Ruminococcus bromii*			↑																		↑	2	0
*Ruminococcus gnavus*			↓																↑			1	1
*Eubacterium hallii*																			↑		↑	2	0
*Eubacterium ventriosum*						↑													↑			2	0
*Faecalibacterium prausnitzii*				↑						↑				↑								3	0
**Proteobacteria**																							
*Sutterella*	↓																			↓		0	2
*Escherichia*		↑																			↓	1	1
*Escheria coli*					↓							↓							↓			0	3
*Escherichia-Shigella*											↓	↓										0	2
*Sutterella wadsworthensis*																			↓		↓	0	2

*Only taxa reported by at least two separate studies are displayed*.

*CD, Crohn's disease; FMT, fecal microbiota transplantation; UC, ulcerative colitis*.

*↑, higher abundance in responders compared with non-responders; ↓, lower abundance in responders compared with non-responders*.

##### Association Between Individual Bacteria and Clinical Phenotypes

A few studies assessed correlations between gut microbiota and clinical outcomes or disease biomarkers (Table 5). *Enterobacteriaceae* (17), *E. coli* (18), an OTU belonging to *F. prausnitzii* (28), taxa belonging to the class Gammaproteobacteria and the order Clostridiales comprising *Ruminococcus gnavus* (39), and engraftment of Proteobacteria and Bacteroidetes (40) were found to be correlated with higher disease severity or relapse in separate studies. In contrast, two studies showed a negative correlation between endoscopic sum score and Bacteroidetes (22), and F-calprotectin levels and α-diversity (28), respectively. Furthermore, three other studies found that certain bacteria benefited the clinical outcome. Li et al. (11) demonstrated that the differences of abundance in *Eggerthella, Lactobacillus*, and *Ruminococcus* between pre- and post-FMT were positively correlated with efficacy. In the trial by Costello et al. (38), increased abundance of *Anaerofilum pentosovorans* and *Bacteroides coprophilus* was strongly associated with disease improvement following FMT. In addition, *Ruminococcaceae, Coprococcus*, and *Desulfovibrio* were associated with the maintenance of remission after FMT (39).

**Table 5:** Correlation between microbiota and clinical phenotypes.

**References**	**Microbial taxa**	**Clinical phenotypes**	**Correlation**
Angelberger et al. (17)	*Enterobacteriaceae*	Mayo score	+
Suskind et al. (18)	*E. coli*	Calprotectin and disease activity	+
Ishikawa et al. (22)	Bacteroidetes	Endoscopic sum score	–
Cold et al. (28)	An OTU belonging to *Faecalibacterium prausnitzii*	SCCAI	+
	α-diversity	F-calprotectin levels	–
Li et al. (11)	The differences of the relative abundance in genera *Eggerthella, Lactobacillus*, and *Ruminococcus* between pre-FMT and 5 days post-FMT	Clinical efficacy	+
Costello et al. (38)	*Anaerofilum pentosovorans, Bacteroides coprophilus*	Disease improvement	+
Sokol et al. (39)	Taxa belonging to Gammaproteobacteria and Clostridiales comprising *Ruminococcus gnavus*	Flare	+
	*Ruminococcaceae, Coprococcus, Desulfovibrio*	Maintenance of remission	+
Kong et al. (40)	Engraftment of Proteobacteria and Bacteroidetes	Relapse	+

*FMT, fecal microbiota transplantation; SCCAI, Simple Clinical Colitis Activity Index. +, positive; –, negative*.

##### Differences in Bacterial Metabolic Pathways or Metabolites

Detailed findings of bacterial metabolic pathways or metabolites are provided in Table 6. Pathways related to increased energy metabolism or components needed for bacterial cell surface or cell walls were increased in responders after FMT compared to non-responders (19), while pathways related to the biosynthesis of Heme, lipopolysaccharide/lipid A, peptidoglycan, ubiquinone and lysine, and oxidative phosphorylation were increased in non-responders (10). Moreover, a study performed by Kong et al. revealed that relapsers after FMT have a depletion in community potential for anaerobic, energy metabolism, NAD biosynthesis, and transfer RNA charging pathways (40). Regarding bacterial metabolites, the metabolomic profile of responders shifts to donors after FMT in the study of Nusbaum et al. and, in particular, fecal butyrate acid increased in responders, which is consistent with the finding by the study of Ohmiya et al. (33). However, fecal butyrate acid and other short-chain fatty acids (SCFA) concentrations were not associated with treatment effect in another study (38).

**Table 6:** Alterations of microbial gene pathways or metabolites.

**References**	**Alterations of microbial gene pathways or metabolites**
Vaughn et al. (19)	↑ in R: Pathways related to energy metabolism or components needed for bacterial cell surface or cell walls (serine and glutamine metabolic pathways, folic acid metabolic pathways, and lipid A biosynthetic pathways)
Nusbaum et al. (27)	Metabolomic profile of R shifts to donors after FMT; ↑ in R: Xanthine, oleic acid, butyric acid; ↓ in R: Putrescine, 5-aminovaleric acid, acetic acid
Fan et al. (29)	↑ in R: taurochenodeoxycholate and taurocholate
Ohmiya et al. (33)	↑ in R of CD: butyrate and secondary bile acids
Paramsothy et al. (10)	↑ in NR: heme biosynthesis, lipopolysaccharide/lipid A biosynthesis, peptidoglycan biosynthesis, ubiquinone and other terpenoid quinine biosynthesis, lysine biosynthesis, and oxidative phosphorylation pathways; ↑ in NR: heme, lysine; ↓ in NR: biotin, dehydrolithocholate
Costello et al. (38)	Stool SCFA concentrations were not associated with treatment effect
Kong et al. (40)	Relapsers had a depletion in community potential for anaerobic, energy metabolism, the NAD biosynthesis and transfer RNA charging pathways

*CD, Crohn's disease; FMT, fecal microbiota transplantation; R, responders; NR, non-responders; SCFA, short chain fatty acids*.

## Discussion

Gut dysbiosis has drawn increasing attention for its role in the pathogenesis of IBD. Numerous studies have described the gut microbial features in patients with IBD (3), thus promoting the development of microbiota-targeted therapeutic methods, such as FMT. Given the heterogeneity of clinical outcomes in individual patients with IBD after receiving FMT, a better understanding of the factors that influence the response to FMT will help to optimize the treatment strategy. In this systematic review, we focused on the microbial distinction between FMT responders and non-responders, and the results showed several convergent findings.

First of all, the delivery route is a significant factor that influences treatment efficacy. The most used route was the colonoscope, while other routes included capsules, nasoduodenal tube, nasojejunal tube, transendoscopic enteral tubing (TET), and retention enema. Previous systemic review and meta-analysis have reported that the remission rate of patients with UC receiving FMT through lower gastrointestinal (GI) administration was much higher than that of upper GI administration (43, 44). It seems that the lower GI route has a trend of superiority over the upper GI route for the treatment of IBD, and this needs to be investigated in further research.

The numbers of infusions and follow-up duration also differed among the studies. There is no uniform conclusion on the lasting time of FMT effect. Li et al. (45) reported that the median time of maintaining clinical response to FMT in 69 patients with CD was 125 days in the first place. Among the 56 patients who received the second FMT, the time of maintaining clinical response was 176.5 days. Their data demonstrated that patients with CD should be given the second course of FMT within 4 months after the first FMT to maintain the clinical benefits of the first FMT.

Stool is a non-standardized material with heterogeneous microbial composition between individual donors, thus the donor stool is a key determinant for a successful FMT. Six of the included studies applied multi-donor stool preparation to increase microbial diversity and the possibility of recipients receiving therapeutically effective donor stool. When analyzing microbial features, we found that the structural difference between responders and donors was larger than that between non-responders and donors. However, responders had a higher increasing degree of microbial similarity to donors than non-responders. This perhaps means that the higher abundance of certain bacterial species in donors is conducive to FMT treatment. By comparing the microbial composition of R-d and NR-d, we observed that R-d had a higher richness and different microbial structure from donors of non-responders in most studies. This further supports the view that successful FMT was highly donor-dependent, and suggests us the necessity to incorporate the analysis of microbiota into the screening of donor stools in the future.

Microbial diversity is a crucial indicator of community stability and function. Decreased diversity was observed in many diseases compared to healthy controls, including IBD (3). In this review, we found that most of the studies reported higher diversity or a greater degree of increased diversity in responders than in non-responders, thus it can be speculated that the effective treatment may be a result of the restoration of microbial homeostasis.

The acquirement of baseline microbial features of patients is needed to predict the FMT outcome. However, only two studies compared the microbiome composition between responders and non-responders. Intriguingly, in addition to the probiotic genera *Bifidobacterium, Fusobacterium*, and *Enterobacteriaceae*, the two potentially pathogenic microorganisms, were also higher in abundance in responders' pre-FMT microbiome. *Fusobacterium* were capable of introducing host inflammatory or tumorigenic responses, predominantly by its unique FadA adhesin (46), and the family *Enterobacteriaceae* was associated with severe infectious diseases (47). These findings were consistent with the higher increasing degree of microbial similarity to donors in responders by Goyal et al. (24), and whether a bigger gap between responders and their donors might result in more effective treatment deserves further investigation.

Regarding the microbial alteration after FMT, we observed some common patterns. Responders presented an increase in relative abundances of SCFA-producing bacteria, such as the genera *Blautia, Faecalibacterium, Eubacterium, Roseburia*, and *Ruminococcus*, all of which were core genera in the healthy population worldwide (48). Among them, the important role of *Faecalibacterium* and *Roseburia* in IBD has been recognized in recent years. It has been generally shown that patients with IBD had a lower abundance of *F. prausnitzii* and, furthermore, active patients had a lower abundance of *F. prausnitzii* than patients in remission (49). In previous preclinical experiments, *F. prausnitzii* has been proven to efficiently alleviate intestinal inflammation, mainly by blocking nuclear factor kappa B (NF-κB) activation and pro-inflammatory cytokine production, and promoting anti-inflammatory IL-10 secretion (50). A recent study revealed that *F. prausnitzii*-derived butyrate exerted an anti-inflammatory effect by upregulating the expression of *Dact3*, a gene involved in the Wnt/JNK pathway (51). *Roseburia* is another butyrate-producing genus, and could also serve as a biomarker for IBD (4). In general, *Roseburia intestinalis* and *R. hominis* are the two most studied species associated with IBD. In our review, however, two studies reported an increase in the abundance of *Roseburia faecis* (17) and *Roseburia inulinivorans* (10) in responders, respectively. Apart from butyrate production, *Roseburia* could also affect the host by its flagellin (52). Furthermore, one study specially focused on the genus *Akkermansia* (35). The positive correlation between the abundance of *Akkermansia* in responders' and donors' demonstrated its successful colonization in the gut. Intriguingly, this study found a co-occurrence relationship between *Akkermansia* and *F. prausnitzii*. This suggests us that the combination of these next-generation probiotics could serve as a supplementary method of FMT, so as to increase the response rate.

The abundance of certain pathogenic bacteria belonging to the phylum Proteobacteria was decreased after FMT in responders. These bacteria included *E. coli* and *Escherichia-Shigella*. *E. coli* has proven to have an abnormal immune and proinflammatory response in IBD (53). In addition, *Enterococcus faecium* V583 could secrete proteases to induce epithelial cell permeability (54), and promote intestinal cytokine expression. The elimination of these potential pathobionts may contribute to an effective response to FMT.

At the metabolic level, two studies reported increased butyrate concentrations in responders, which was consistent with the enrichment of butyrate-producing taxa in the studies. We also found an alteration of bile acids enriched in responders. Patients with IBD have reduced levels of lithocholic acid and deoxycholic acid (main secondary bile acids, SBA), and SBA supplementation could reduce intestinal inflammation (55). Although the results were divergent, microbial functional content analysis revealed differentially abundant pathways involved in energy metabolism and biosynthesis of virulence factors. Metabolic and functional alterations need to be further unraveled as studies on them are scarce.

Given the lack of a standardized procedure for FMT in patients with IBD, this systematic review had several limitations. Firstly, almost all studies only analyzed the microbiome from stool samples. However, mucosal microbiota may play a more important role due to their direct crosstalk with intestinal tissues. Hence, more studies concerning the mucosal microbiome associated with the response to FMT should be conducted in future research. Secondly, the different methods used for microbiota detection may lead to different conclusions about the microbial alteration. For example, the relative abundance of the potential probiotic species, *B. ovatus*, was reported to be increased in responders in two studies and decrease in the other two studies. These four studies used HITChip (37), whole-genome shotgun sequencing (19), pyrosequencing (17), and 16S amplicon sequencing (16), respectively, to assess the microbiota.

## Conclusions

**Figure 2:**
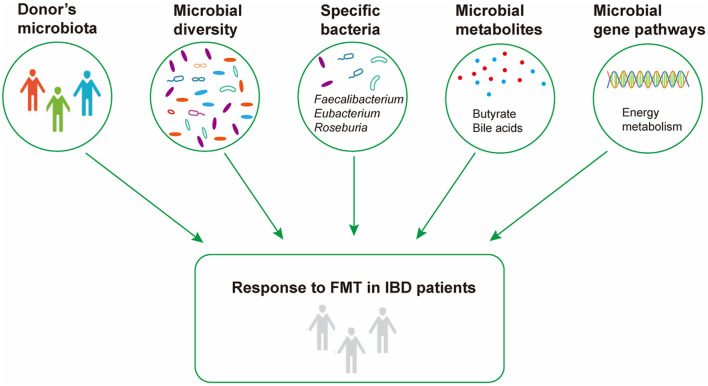
Microbial factors influencing response to fecal microbiota transplantation in inflammatory bowel disease.

In conclusion, our systematic review revealed that the response to FMT was associated with gut microbiota and their metabolites, and the different results among different studies were probably attributed to the methodology of FMT, such as ways of delivery and number of infusions (Figure 2). The pre-FMT microbial features of recipients, the comparison of pre- and post-FMT microbiota, and the relationship between recipients and donors at baseline should be further investigated using uniform and standardized methods to develop the gut microbiome as a new biomarker for predicting the treatment effect of FMT, and perhaps presupplementation or depletion of specific bacterial taxa or metabolic molecule could enhance the curative effect of FMT.

## Data Availability Statement

The original contributions presented in the study are included in the article/Supplementary Material, further inquiries can be directed to the corresponding author/s.

## Author Contributions

JZ and YG: concept and design, database searching, literature screening, data interpretation, manuscript drafting, and final approval of manuscript. LD: concept and design, data interpretation, critical revision of manuscript, and final approval of manuscript. All authors contributed to the article and approved the submitted version.

## Funding

This work was supported by National Natural Science Foundation of China (Grant No. 82000510) and National Key R&D Program of China (Grant No. 2019YFA0905600).

## Conflict of Interest

The authors declare that the research was conducted in the absence of any commercial or financial relationships that could be construed as a potential conflict of interest.

## Publisher's Note

All claims expressed in this article are solely those of the authors and do not necessarily represent those of their affiliated organizations, or those of the publisher, the editors and the reviewers. Any product that may be evaluated in this article, or claim that may be made by its manufacturer, is not guaranteed or endorsed by the publisher.

## References

[1] Ng SC Shi HY Hamidi N Underwood FE Tang W Benchimol EI . Worldwide incidence and prevalence of inflammatory bowel disease in the[[sp]] 2[[/sp]]1st century: a systematic review of population-based studies. Lancet. (2018) 390:2769–78. 10.1016/S0140-6736(17)32448-0 29050646

[2] Chang JT. Pathophysiology of inflammatory bowel diseases. N Engl J Med. (2020) 383:2652–64. 10.1056/NEJMra2002697 33382932

[3] Pittayanon R Lau JT Leontiadis GI Tse F Yuan Y Surette M . Differences in gut microbiota in patients with vs without inflammatory bowel diseases: a systematic review. Gastroenterology. (2019) 157:97–108. 10.1053/j.gastro.2019.03.049 30940523

[4] Machiels K Joossens M Sabino J De Preter V Arijs I Eeckhaut V . A decrease of the butyrate-producing species Roseburia hominis and *Faecalibacterium prausnitzii* defines dysbiosis in patients with ulcerative colitis. Gut. (2014) 63:1275–83. 10.1136/gutjnl-2013-304833 24021287

[5] Lavelle A Sokol H. Gut microbiota-derived metabolites as key actors in inflammatory bowel disease. Nat Rev Gastroenterol Hepatol. (2020) 17:223–37. 10.1038/s41575-019-0258-z 32076145

[6] Jakubczyk D Leszczynska K Gorska S. The effectiveness of probiotics in the treatment of inflammatory bowel disease (IBD)-a critical review. Nutrients. (2020) 12:1973. 10.3390/nu12071973 32630805 PMC7400428

[7] Glassner KL Abraham BP Quigley EMM. The microbiome and inflammatory bowel disease. J Allergy Clin Immunol. (2020) 145:16–27. 10.1016/j.jaci.2019.11.003 31910984

[8] Mcdonald LC Gerding DN Johnson S Bakken JS Carroll KC Coffin SE . Clinical practice guidelines for clostridium difficile infection in adults and children: 2017 update by the Infectious Diseases Society of America (IDSA) and Society for Healthcare Epidemiology of America (SHEA). Clin Infect Dis. (2018) 66:987–94. 10.1093/cid/ciy149 29562266

[9] Costello SP Soo W Bryant RV Jairath V Hart AL Andrews JM. Systematic review with meta-analysis: faecal microbiota transplantation for the induction of remission for active ulcerative colitis. Aliment Pharmacol Ther. (2017) 46:213–24. 10.1111/apt.14173 28612983

[10] Paramsothy S Nielsen S Kamm MA Deshpande NP Faith JJ Clemente JC . Specific bacteria and metabolites associated with response to fecal microbiota transplantation in patients with ulcerative colitis. Gastroenterology. (2019) 156:1440–54 e2. 10.1053/j.gastro.2018.12.001 30529583

[11] Li QQ Ding X Liu KJ Marcella C Liu XL Zhang T . Fecal microbiota transplantation for ulcerative colitis: the optimum timing and gut microbiota as predictors for long-term clinical outcomes. Clin Transl Gastroenterol. (2020) 11:e00224. 10.14309/ctg.0000000000000224 32955197 PMC7431231

[12] Moher D Liberati A Tetzlaff J Altman DG Group P. Preferred reporting items for systematic reviews and meta-analyses: the PRISMA statement. BMJ. (2009) 339:b2535. 10.1136/bmj.b2535 19622551 PMC2714657

[13] Higgins JP Altman DG Gotzsche PC Juni P Moher D Oxman AD . The Cochrane Collaboration's tool for assessing risk of bias in randomised trials. BMJ. (2011) 343:d5928. 10.1136/bmj.d5928 22008217 PMC3196245

[14] Kao D Hotte N Gillevet P Madsen K. Fecal microbiota transplantation inducing remission in Crohn's colitis and the associated changes in fecal microbial profile. J Clin Gastroenterol. (2014) 48:625–8. 10.1097/MCG.0000000000000131 24667590

[15] Shimizu H Arai K Abe J Nakabayashi K Yoshioka T Hosoi K . Repeated fecal microbiota transplantation in a child with ulcerative colitis. Pediatr Int. (2016) 58:781–5. 10.1111/ped.12967 27324973

[16] Quagliariello A Del Chierico F Reddel S Russo A Onetti Muda A D'argenio P . Fecal microbiota transplant in two ulcerative colitis pediatric cases: gut microbiota and clinical course correlations. Microorganisms. (2020) 8:1486. 10.3390/microorganisms8101486 32992653 PMC7599854

[17] Angelberger S Reinisch W Makristathis A Lichtenberger C Dejaco C Papay P . Temporal bacterial community dynamics vary among ulcerative colitis patients after fecal microbiota transplantation. Am J Gastroenterol. (2013) 108:1620–30. 10.1038/ajg.2013.257 24060759

[18] Suskind DL Brittnacher MJ Wahbeh G Shaffer ML Hayden HS Qin X . Fecal microbial transplant effect on clinical outcomes and fecal microbiome in active Crohn's disease. Inflamm Bowel Dis. (2015) 21:556–63. 10.1097/MIB.0000000000000307 25647155 PMC4329080

[19] Vaughn BP Vatanen T Allegretti JR Bai AP Xavier RJ Korzenik J . Increased intestinal microbial diversity following fecal microbiota transplant for active Crohn's disease. Inflamm Bowel Dis. (2016) 22:2182–90. 10.1097/MIB.0000000000000893 27542133 PMC4995064

[20] Vermeire S Joossens M Verbeke K Wang J Machiels K Sabino J . Donor species richness determines faecal microbiota transplantation success in inflammatory bowel disease. J Crohns Colitis. (2016) 10:387–94. 10.1093/ecco-jcc/jjv203 26519463 PMC4946755

[21] Jacob V Crawford C Cohen-Mekelburg S Viladomiu M Putzel GG Schneider Y . Single delivery of high-diversity fecal microbiota preparation by colonoscopy is safe and effective in increasing microbial diversity in active ulcerative colitis. Inflamm Bowel Dis. (2017) 23:903–11. 10.1097/MIB.0000000000001132 28445246 PMC6159890

[22] Ishikawa D Sasaki T Osada T Kuwahara-Arai K Haga K Shibuya T . Changes in intestinal microbiota following combination therapy with fecal microbial transplantation and antibiotics for ulcerative colitis. Inflamm Bowel Dis. (2017) 23:116–25. 10.1097/MIB.0000000000000975 27893543

[23] Nishida A Imaeda H Ohno M Inatomi O Bamba S Sugimoto M . Efficacy and safety of single fecal microbiota transplantation for Japanese patients with mild to moderately active ulcerative colitis. J Gastroenterol. (2017) 52:476–82. 10.1007/s00535-016-1271-4 27730312

[24] Goyal A Yeh A Bush BR Firek BA sSiebold LM Rogers MB . Safety, Clinical response, and microbiome findings following fecal microbiota transplant in children with inflammatory bowel disease. Inflamm Bowel Dis. (2018) 24:410–21. 10.1093/ibd/izx035 29361092

[25] Karakan T Karataş A. Donor microbiota as a determinant factor for response to FMT in patients with ulcerative colitis. United Eur Gastroenterol J. (2018) 6:A133–4.

[26] Kump P Wurm P Gröchenig HP Wenzl H Petritsch W Halwachs B . The taxonomic composition of the donor intestinal microbiota is a major factor influencing the efficacy of faecal microbiota transplantation in therapy refractory ulcerative colitis. Aliment Pharmacol Ther. (2018) 47:67–77. 10.1111/apt.14387 29052237 PMC5765501

[27] Nusbaum DJ Sun F Ren J Zhu Z Ramsy N Pervolarakis N . Gut microbial and metabolomic profiles after fecal microbiota transplantation in pediatric ulcerative colitis patients. FEMS Microbiol Ecol. (2018) 94:fiy133. 10.1093/femsec/fiy133 30010747 PMC6454419

[28] Cold F Browne PD Günther S Halkjaer SI Petersen AM Al-Gibouri Z . Multidonor FMT capsules improve symptoms and decrease fecal calprotectin in ulcerative colitis patients while treated–an open-label pilot study. Scand J Gastroenterol. (2019) 54:289–96. 10.1080/00365521.2019.1585939 30946615

[29] Fan Y Chen Q Zhang B Chen Z Huang Q Xu H . Effect of multidonor intensive fecal microbiota transplantation by capsules for active uncreative colitis: a prospective trial. Gut. (2019) 68:A109–10. 10.1136/gutjnl-2019-IDDFAbstracts.21028850992

[30] Gogokhia L Crawford CV Lima SF Viladomiu M Jacob V Scherl E . Transferable IGA-reactive microbiota stratify clinical response to fmt for ulcerative colitis. Gastroenterology. (2019) 156:S239. 10.1016/S0016-5085(19)37397-4

[31] Gutin L Piceno Y Fadrosh D Lynch K Zydek M Kassam Z . Fecal microbiota transplant for Crohn disease: a study evaluating safety, efficacy, and microbiome profile. United Eur Gastroenterol J. (2019) 7:807–14. 10.1177/2050640619845986 31316785 PMC6620877

[32] Chen HT Huang HL Xu HM Luo QL He J Li YQ . Fecal microbiota transplantation ameliorates active ulcerative colitis. Exp Ther Med. (2020) 19:2650–60. 10.3892/etm.2020.8512 32256746 PMC7086197

[33] Ohmiya N Osaki H Jodai Y Koyama K Maeda K Omori T . Changes in fecal microbiota, short chain fatty acids, and bile acids after fecal microbiota transplantation for recurrent clostridioides difficile infection, ulcerative colitis, and Crohn's disease. Gastroenterology. (2020) 158:S483. 10.1016/S0016-5085(20)31885-0

[34] Schierova D Brezina J Mrazek J Fliegerova KO Kvasnova S Bajer L . Gut microbiome changes in patients with active left-sided ulcerative colitis after fecal microbiome transplantation and topical 5-aminosalicylic acid therapy. Cells. (2020) 9:2283. 10.3390/cells9102283 33066233 PMC7602113

[35] Zhang T Li P Wu X Lu G Marcella C Ji X . Alterations of Akkermansia muciniphila in the inflammatory bowel disease patients with washed microbiota transplantation. Appl Microbiol Biotechnol. (2020). 104:10203–15. 10.1007/s00253-020-10948-7 33064186

[36] Rossen NG Fuentes S Van Der Spek MJ Tijssen JG Hartman JH Duflou A . Findings from a randomized controlled trial of fecal transplantation for patients with ulcerative colitis. Gastroenterology. (2015) 149:110–8.e4. 10.1053/j.gastro.2015.03.045 25836986

[37] Fuentes S Rossen NG Van Der Spek MJ Hartman JHA Huuskonen L Korpela K . Microbial shifts and signatures of long-term remission in ulcerative colitis after faecal microbiota transplantation. Isme J. (2017) 11:1877–89. 10.1038/ismej.2017.44 28398347 PMC5520032

[38] Costello SP Hughes PA Waters O Bryant RV Vincent AD Blatchford P . Effect of fecal microbiota transplantation on 8-week remission in patients with ulcerative colitis: a randomized clinical trial. JAMA. (2019) 321:156–64. 10.1001/jama.2018.20046 30644982 PMC6439766

[39] Sokol H Landman C Seksik P Berard L Montil M Nion-Larmurier I . Fecal microbiota transplantation to maintain remission in Crohn's disease: a pilot randomized controlled study. Microbiome. (2020) 8:12. 10.1186/s40168-020-0792-5 32014035 PMC6998149

[40] Kong L Lloyd-Price J Vatanen T Seksik P Beaugerie L Simon T . Linking strain engraftment in fecal microbiota transplantation with maintenance of remission in Crohn's disease. Gastroenterology. (2020). 159:2193–202. 10.1053/j.gastro.2020.08.045 32860788 PMC7725862

[41] Zhang T Lu G Zhao Z Liu Y Shen Q Li P . Washed microbiota transplantation vs. manual fecal microbiota transplantation: clinical findings, animal studies and *in vitro* screening. Protein Cell. (2020) 11:251–66. 10.1007/s13238-019-00684-8 31919742 PMC7093410

[42] Paramsothy S Kamm MA Kaakoush NO Walsh AJ Van Den Bogaerde J Samuel D . Multidonor intensive faecal microbiota transplantation for active ulcerative colitis: a randomised placebo-controlled trial. Lancet. (2017) 389:1218–28. 10.1016/S0140-6736(17)30182-4 28214091

[43] Shi Y Dong Y Huang W Zhu D Mao H Su P. Fecal microbiota transplantation for ulcerative colitis: a systematic review and meta-analysis. PLoS ONE. (2016) 11:e0157259. 10.1371/journal.pone.0157259 27295210 PMC4905678

[44] Paramsothy S Paramsothy R Rubin DT Kamm MA Kaakoush NO Mitchell HM . Faecal microbiota transplantation for inflammatory bowel disease: a systematic review and meta-analysis. J Crohns Colitis. (2017) 11:1180–99. 10.1093/ecco-jcc/jjx063 28486648

[45] Li P Zhang T Xiao Y Tian L Cui B Ji G . Timing for the second fecal microbiota transplantation to maintain the long-term benefit from the first treatment for Crohn's disease. Appl Microbiol Biotechnol. (2019) 103:349–60. 10.1007/s00253-018-9447-x 30357440 PMC6311185

[46] Han YW. Fusobacterium nucleatum: a commensal-turned pathogen. Curr Opin Microbiol. (2015) 23:141–7. 10.1016/j.mib.2014.11.013 25576662 PMC4323942

[47] Janda JM Abbott SL. The changing face of the family Enterobacteriaceae (Order: “Enterobacterales”): new members, taxonomic issues, geographic expansion, and new diseases and disease syndromes. Clin Microbiol Rev. (2021) 34:e00174–20. 10.1128/CMR.00174-20 33627443 PMC8262773

[48] Dehingia M Devi KT Talukdar NC Talukdar R Reddy N Mande SS . Gut bacterial diversity of the tribes of India and comparison with the worldwide data. Sci Rep. (2015) 5:18563. 10.1038/srep18563 26689136 PMC4686986

[49] Zhao H Xu H Chen S He J Zhou Y Nie Y. Systematic review and meta-analysis of the role of Faecalibacterium prausnitzii alteration in inflammatory bowel disease. J Gastroenterol Hepatol. (2021) 36:320–8. 10.1111/jgh.15222 32815163

[50] Sokol H Pigneur B Watterlot L Lakhdari O Bermudez-Humaran LG Gratadoux JJ . Faecalibacterium prausnitzii is an anti-inflammatory commensal bacterium identified by gut microbiota analysis of Crohn disease patients. Proc Natl Acad Sci USA. (2008) 105:16731–6. 10.1073/pnas.0804812105 18936492 PMC2575488

[51] Lenoir M Martin R Torres-Maravilla E Chadi S Gonzalez-Davila P Sokol H . Butyrate mediates anti-inflammatory effects of Faecalibacterium prausnitzii in intestinal epithelial cells through Dact3. Gut Microbes. (2020) 12:1–16. 10.1080/19490976.2020.1826748 33054518 PMC7567499

[52] Seo B Jeon K Moon S Lee K Kim WK Jeong H . Roseburia spp. abundance associates with alcohol consumption in humans and its administration ameliorates alcoholic fatty liver in mice. Cell Host Microbe. (2020) 27:25–40 e6. 10.1016/j.chom.2019.11.001 31866426

[53] Petersen AM Halkjaer SI Gluud LL. Intestinal colonization with phylogenetic group B2 Escherichia coli related to inflammatory bowel disease: a systematic review and meta-analysis. Scand J Gastroenterol. (2015) 50:1199–207. 10.3109/00365521.2015.1028993 25910859

[54] Maharshak N Huh EY Paiboonrungruang C Shanahan M Thurlow L Herzog J . Enterococcus faecalis gelatinase mediates intestinal permeability via protease-activated receptor 2. Infect Immun. (2015) 83:2762–70. 10.1128/IAI.00425-15 25916983 PMC4468563

[55] Sinha SR Haileselassie Y Nguyen LP Tropini C Wang M Becker LS . Dysbiosis-induced secondary bile acid deficiency promotes intestinal inflammation. Cell Host Microbe. (2020) 27:659–70 e5. 10.1016/j.chom.2020.01.021 32101703 PMC8172352

