# Supplementary material for: Features of Gut Microbiome Associated With Responses to Fecal Microbiota Transplantation for Inflammatory Bowel Disease: A Systematic Review
Source: Front Med (Lausanne). 2022 May 26;9:773105. doi: 10.3389/fmed.2022.773105 (PMC9198717; doi:10.3389/fmed.2022.773105)
Supplement: Supplementary file 1 [file Data_Sheet_1.docx]

**Search strategy:**

(((FMT) OR (bacteriotherapy)) OR (((fecal) OR (faecal) OR (stool) OR (feces) OR (faeces) OR (microbiota) OR (microflora) OR (fecal flora)

OR (faecal flora) OR (fecal microbiota) OR (feacal microbiota)) AND ((transplant*) OR (transfusion) OR (implant*) OR (instillation) OR

(donor) OR (enema) OR (reconstitution) OR (infusion) OR (transfer)))) AND ((Crohns disease) OR (Crohn's Disease) OR (Crohn Disease) OR

(Ulcerative Colitis) OR (Inflammatory bowel disease) OR (UC) OR (IBD) OR (CD))Table S1. Quality assessment of cohort studies

| Author | | Representativeness of the exposed cohort to average active IBD patient | | Selection of the non exposed cohort | | Ascertainment of FMT exposure | Demonstration that outcome of interest was not present at start of study | | | Comparability of disease severity | | Comparability of disease duration or medications | | Assessment of outcome | | Was follow-up long enough for outcomes to occur | | Adequacy of follow up of cohorts | | NOS  Total |
| --- | --- | --- | --- | --- | --- | --- | --- | --- | --- | --- | --- | --- | --- | --- | --- | --- | --- | --- | --- | --- |
| Angelberger et al.^17^ | | 0 | | 0 | | 1 | 1 | | | 0 | | 0 | | 1 | | 1 | | 1 | | 5 |
| Suskind et al.^18^ | | 1 | | 0 | | 1 | 1 | | | 0 | | 0 | | 1 | | 1 | | 1 | | 6 |
| Vaughn et al.^25^ | | 1 | | 0 | | 1 | 1 | | | 0 | | 0 | | 1 | | 1 | | 1 | | 6 |
| Vermeire et al.^30^ | | 0 | | 0 | | 1 | 1 | | | 0 | | 0 | | 1 | | 1 | | 1 | | 5 |
| Jacob et al.^32^ | | 1 | | 0 | | 1 | 1 | | | 0 | | 0 | | 1 | | 1 | | 1 | | 6 |
| Ishikawa et al.^21^ | | 1 | | 1 | | 1 | 1 | | | 1 | | 1 | | 1 | | 1 | | 1 | | 9 |
| Nishida et al.^33^ | | 1 | | 0 | | 1 | 1 | | | 0 | | 0 | | 1 | | 1 | | 1 | | 6 |
| Goyal et al.^23^ | | 1 | | 0 | | 1 | 1 | | | 0 | | 0 | | 1 | | 1 | | 1 | | 6 |
| Karakan et al.^31^ | | 0 | | 0 | | 1 | 1 | | | 0 | | 0 | | 1 | | 1 | | 1 | | 5 |
| Kump et al.^22^ | | 1 | | 1 | | 1 | 1 | | | 1 | | 1 | | 1 | | 1 | | 1 | | 9 |
| Nusbaum et al.^26^ | | 1 | | 0 | | 1 | 1 | | | 0 | | 0 | | 1 | | 1 | | 1 | | 6 |
| Cold et al.^24^ | | 1 | | 0 | | 1 | 1 | | | 0 | | 0 | | 1 | | 1 | | 1 | | 6 |
| Fan et al.^27^ | | 0 | | 0 | | 1 | 1 | | | 0 | | 0 | | 1 | | 1 | | 1 | | 5 |
| Gogokhia et al.^35^ | | 0 | | 0 | | 1 | 1 | | | 0 | | 0 | | 1 | | 1 | | 1 | | 5 |
| Gutin et al.^34^ | | 1 | | 0 | | 1 | 1 | | | 0 | | 0 | | 1 | | 1 | | 1 | | 6 |
| Chen et al.^39^ | | 1 | | 0 | | 1 | 1 | | | 0 | | 0 | | 1 | | 1 | | 1 | | 6 |
| Table S1. Continued | | | | | | | | | | | | | | | | | | | | |
| Author | Representativeness of the exposed cohort to average active IBD patient | | Selection of the non exposed cohort | | Ascertainment of FMT exposure | | | Demonstration that outcome of interest was not present at start of study | Comparability of disease severity | | Comparability of disease duration or medications | | Assessment of outcome | | Was follow-up long enough for outcomes to occur | | Adequacy of follow up of cohorts | | NOS  Total | |
| Li et al.^11^ | | 0 | | 0 | | 1 | 1 | | | 0 | | 0 | | 1 | | 1 | | 1 | | 5 |
| Ohmiya et al.^36^ | | 0 | | 0 | | 1 | 1 | | | 0 | | 0 | | 1 | | 1 | | 1 | | 5 |
| Schierová et al.^54^ | | 1 | | 1 | | 1 | 1 | | | 1 | | 1 | | 1 | | 1 | | 1 | | 9 |
| Zhang et al.^19^ | | 0 | | 0 | | 1 | 1 | | | 0 | | 0 | | 1 | | 1 | | 1 | | 5 |

NOS, Newcastle Ottawa Scale.

| Author | Random sequence generation | Allocation concealment | Blinding of participants and personnel | Blinding of outcome assessment | Incomplete outcome data | Selective reporting |
| --- | --- | --- | --- | --- | --- | --- |
| Rossen et al.^29^ | Unclear | Unclear | Low | Low | Low | Low |
| Fuentes et al.^38^ | Unclear | Unclear | Low | Low | Low | Low |
| Paramsothy et al.  (2017^37^, 2019^49^) | Low | Low | Low | Low | Low | Low |
| Costello et al.^42^ | Low | Low | Low | Low | Low | Low |
| Sokol et al.^40^  Kong et al.^41^ | Low | Low | High | High | Low | Low |

Table S2. Risk of bias assessment for RCT

Table S3. Characteristics of the included studies

| Author | Year | Country | Study type | IBD type | Patients (n) | Age | Gender  (Male %) | Disease severity | Treatment before FMT |
| --- | --- | --- | --- | --- | --- | --- | --- | --- | --- |
| Kao et al.^14^ | 2014 | Canada | Case report | CD | 1 | 27 | 1 | HBI=12 | Fistulotomy, ciprofloxacin, metronidazole, mesalamine, prednisone, azathioprine |
| Shimizu et al.^15^ | 2016 | Japan | Case report | UC | 1 | 11 | 0 | Refractory, Steroid dependent | 5-ASA, corticosteroids, tacrolimus, infliximab |
| Quagliariello et al.^16^ | 2020 | Italy | Case series | UC | 2 | 15, 16 | NR | Mild to moderate (Mayo score 0-2, PUCAI 3-35) | Mesalazine, azathioprine |
| Angelberger et al. ^17^ | 2013 | Austria | Cohort | UC | 5 | 22-51 | 3 | Moderate to severe (Mayo score ≥ 6) | Infliximab, golimumab, adalimumab, cyclosporine, thiopurine, methotrexate, steroids |
| Suskind et al.^18^ | 2015 | America | Cohort | CD | 9 | 16.2±2.9 | 5 | Mild to moderate (PUCAI 10-29) | Methotrexate, azathioprine, mercaptopurine, mesalamine |
| Vaughn et al.^25^ | 2016 | America | Cohort | CD | 19 | 36±12.3 | 12 (63%) | HBI ≥ 5 | Mesalamine, steroids, immunomodulators |
| Vermeire et al.^30^ | 2016 | Belgium | Cohort | UC (n=8)  CD (n=6) | 14 | 26-57 | 5 | Intractable, CDAI median 290; Mayo score median 8.5 | 5-ASA, steroids, azathioprine, infliximab |
| Jacob et al.^32^ | 2017 | America | Cohort | UC | 20 | 38.4±12.6 | 12 (60%) | Mayo score ≥ 3 and endoscopic subscore ≥ 1 | Corticosteroids, mesalamine, anti-TNFα, vedolizumab, thiopurines |
| Ishikawa et al.^21^ | 2017 | Japan | Cohort | UC | 17 | 40.4±14.2 | 13 (76%) | CAI ≥ 5 or with Mayo endoscopic score ≥ 1 | 5-ASA, Corticosteroids, anti-TNF, immunosuppressants, tacrolimus,  apheresis, Chinese herbal medicine |
| Nishida et al.^33^ | 2017 | Japan | Cohort | UC | 41 | 39.6 ± 16.9 | 28 | Mild to moderate (Mayo score 3-9, and Mayo endoscopic score ≥ 1) | 5-ASA, glucocorticoids, immunomodulator, anti-TNF drugs |
| Table S3. Continued | | | | | | | | | |
| Author | Year | Country | Study type | IBD type | Patients (n) | Age | Gender  (Male %) | Disease severity | Treatment before FMT |
| Goyal et al.^23^ | 2018 | America | Cohort | CD (n=7) UC(n=12)  IC (n=2) | 21 | 8-21 (median 12) | 12 | Mild to moderate (PCDAI 10–40, PUCAI 10–64) | Mesalamine, corticosteroids, immunomodulators, biologicals |
| Karakan et al.^31^ | 2018 | Turkey | Cohort | UC | 50 | NR | NR | steroid resistant or dependent | NR |
| Kump et al.^22^ | 2018 | Austria | Cohort | UC | 17 | 44±18 | 14 (82%) | Mayo score ≥ 4 and endoscopic subscore ≥ 1 | Mesalazine, immunosuppressants, anti-TNF, corticosteroids |
| Nusbaum et al.^26^ | 2018 | America | Cohort | UC | 7 | 7-20 | NR | Mild to moderate (PUCAI 15-65) | NR |
| Cold et al.^24^ | 2019 | Denmark | Cohort | UC | 7 | 27-50 (median 38) | 5/7 | SCCAI 4-10 | 5-ASA, corticosteroids, thiopurines, biologicals |
| Fan et al.^27^ | 2019 | China | Cohort | UC | 28 | NR | NR | Mayo score 4-11 | NR |
| Gogokhia et al.^35^ | 2019 | America | Cohort | UC | NR | NR | NR | NR | NR |
| Gutin et al.^34^ | 2019 | America | Cohort | CD | 10 | 42±15 | 5 (50%) | HBI ≥ 3 | Steroid, biologic |
| Chen et al.^39^ | 2020 | China | Cohort | UC | 44 | 44.4±15.5 | 25 (57%) | Mild to moderate  (Mayo score 3‑10) | 5-ASA, immunomodulator, steroids |
| Li et al.^11^ | 2020 | China | Cohort | UC | 202 (26 lost),122 analyzed, 22 stool collected | Median 36 | 71 (58.2%) | Mild to severe (Mayo score 3‑12) | Mesalamine, corticosteroids, cyclosporine, azathioprine, anti-TNF |
| Ohmiya et al.^36^ | 2020 | Japan | Cohort | UC (n=28) CD (n=4) | 32 | NR | NR | NR | NR |
| Table S3. Continued | | | | | | | | | |
| Author | Year | Country | Study type | IBD type | Patients (n) | Age | Gender  (Male %) | Disease severity | Treatment before FMT |
| Schierová et al.^54^ | 2020 | Czech Republic | Cohort | UC | 8 | 28-62 (mean 37.5) | 4 (50%) | Mayo score < 10, Endoscopic Mayo score ≥ 2 | Thiopurines, mesalamine |
| Zhang et al.^19^ | 2020 | China | Cohort | UC (n=43) CD (n=57) | 100 | UC: 35.5±12.2；CD: 33.8±16.0 | UC: 24 (55.8%), CD: 34/57 (59.6%) | Mild to severe  (UC: Mayo score 3-12; CD HBI＞5) | Mesalamine, steroids, immunosuppressant, anti-TNFα, traditional Chinese medicine |
| Rossen et al.^29^ | 2015 | Netherlands | RCT | UC | 48  (FMT 23, control 25) | Median: FMT 40, control 41 | FMT: 11 (47.8%); control: 11 (44%) | Mild to moderate  (SCCAI 4-11) | Mesalamine, corticosteroids, immunosuppressants, loperamine, anti-TNF |
| Fuentes et al.^38^ | 2017 | Netherlands | RCT | UC | 34  (responders 12, non-responders 22) | Median: responders 44, non-responders 40.5 | responders 5 (41.6%), non-responders 11 (50%) | Mild to moderate  (SCCAI 4-11) | Mesalamine, corticosteroids, immunosuppressants |
| Paramsothy et al.^37^ | 2017 | Australia | RCT | UC | 81  (FMT 41, control 40) | Median: FMT 35.6, control 35.4 | FMT: 22 (54%); control: 25 (63%) | Mayo score 4-10 | 5-ASA, immunomodulator, steroids, anti-TNF and other biological therapy |
|  | | | | | | | | | |
| Table S3. Continued | | | | | | | | | |
| Author | Year | Country | Study type | IBD type | Patients (n) | Age | Gender  (Male %) | Disease severity | Treatment before FMT |
| Paramsothy et al.^49^ | 2019 | Australia | RCT | UC | 70 analyzed for microbiota | NR | NR | NR | NR |
| Costello et al.^42^ | 2019 | Australia | RCT | UC | 73  (FMT 38, control 35) | Median: FMT 38.5, control 35 | FMT: 20 (53%); control: 20 (57%) | Mayo score 3-10 with endoscopic subscore ≥ 2 | Steroids, 5-ASA, immunomodulator (azathioprine or 6-mercaptopurine), biologics (infliximab or vedolizumab) |
| Sokol et al.^40^  Kong et al.^41^ | 2020 | France | RCT | CD | 17  (FMT 8, control 9) | Median: FMT 31.5, control 34.0 | FMT: 5 (62.5%); control: 4 (44.4%) | HBI < 5 following corticosteroids | Corticosteroids, azathioprine, anti-TNF and other biological |

5-ASA: 5- aminosalicylic acid; CAI, Lichtiger’s Clinical Activity Index ; CD, Crohn’s disease; CDAI, Crohn’s Disease Activity Index; FMT, fecal microbiota transplantation; HBI, Harvey–Bradshaw Index; IBD, inflammatory bowel disease; IC, indeterminate colitis; NR, not recorded; PCDAI, Paediatric Crohn’s Disease Activity Index; PUCAI, Paediatric Ulcerative Colitis Activity Index; RCT, randomized controlled trial; SCCAI, Simple Clinical Colitis Activity Index; TNF, tumor necrosis factor; UC, ulcerative colitis.

Table S4. Clinical outcome assessment of FMT

| Author | Follow-up | Def of  clinical response | Def of  clinical remission | Def of  endoscopic remission | Clinical  response | Clinical  remission | Endoscopic remission |
| --- | --- | --- | --- | --- | --- | --- | --- |
| Kao et al.^14^ | 4 weeks | - | HBI = 0 | NR | - | 1 | 1 |
| Shimizu et al.^15^ | 40 weeks | - | PUCAI = 0 | NR | - | 1 | 0 |
| Quagliariello et al.^16^ | 16 weeks | - | NR | - | - | 1/2 (50%) | - |
| Angelberger et al.^17^ | 12 weeks | Mayo score reduce by ≥ 3  and 30% | Mayo score ≤ 2 | - | 1/5 (20%) | 0 | - |
| Suskind et al.^18^ | 12 weeks | - | PUCAI＜10 | - | - | 5/9 (55.6%) | - |
| Vaughn et al.^25^ | 12 weeks | HBI reduce by ≥ 3 | HBI＜5 | - | 6/19 (31.6%) | 10/19 (52.6%, week 4) | - |
| Vermeire et al.^30^ | 24 weeks | NR | - | UC: Mayo endoscopic subscore ≤ 1;  CD: SES-CD <3 | UC 3/8 (37.5%), CD 1/5 (20%) | - | UC 2/8 (25%), CD 0/6 |
| Jacob et al.^32^ | 12 weeks | Mayo score reduce by ≥ 3 and a bleeding subscore ≤ 1 | Mayo score ≤ 2 and no subscore＞1 | Endoscopy subscore = 0 | 7/20 (35%) | 3/20 (15%) | 2/20 (10%) |
| Ishikawa et al.^21^ | 4 weeks | CAI ≤ 10 and reduce by ≥ 3 | CAI ≤ 3 | NR | 14/17 (82.3%) | 9/17 (53.0%) | NR |
| Table S4. Continued |  |  |  |  |  |  |  |
| Author | Follow-up | Def of  clinical response | Def of  clinical remission | Def of  endoscopic remission | Clinical  response | Clinical  remission | Endoscopic remission |
| Nishida et al.^33^ | 8 weeks | Mayo score reduce by ≥ 3, or Mayo clinical score reduce by ≥ 2, with rectal bleeding subscore reduce by ≥ 1 | Mayo score ≤ 2, with no subscore＞1 | - | 11/41 (26.8%) | 0 | - |
| Goyal et al.^23^ | 6 months | PUCAI reduce by 15, or PCDAI reduce by 12.5 | PUCAI/PCDAI=0 | - | UC/IC 3/14 (21.4%), CD 3/7 (43%) | UC/IC 0/14, CD 2/7 (28.6%) | - |
| Karakan et al.^31^ | NR | NR | NR | - | 16/50 (32%) | 4/50 (8%) | - |
| Kump et al.^22^ | 3 months | Mayo score reduce by ≥ 3 | Mayo score ≤ 2 | Mayo endoscopic score ≤ 1 | 10/17 (59%) | 4/17 (24%) | 4/17 (24%) |
| Nusbaum et al.^26^ | 4 weeks | PUCAI ≤ 15 | - | - | 4/7 (57.1%) | - | - |
| Cold et al.^24^ | 6 months | SCCAI reduce by ≥ 1.5  Responder: FCP reduce by ≥ 500mg/kg at 4 weeks | SCCAI ≤ 2 | - | 7 /7 (100%, 4 weeks) | 5/7 (71.4%, 4 weeks) | - |
| Fan et al.^27^ | NR | - | NR | Mucosal healing | - | 16/28 (57.14%) | 11/22 (50%) |
| Gogokhia et al.^35^ | NR | NR | NR | NR | NR | NR | NR |
| Gutin et al.^34^ | 12 months | HBI reduce by ≥ 3 | HBI＜3 | - | 3/10 (30%) | 1/10 (10%) | - |
| Table S4. Continued |  |  |  |  |  |  |  |
| Author | Follow-up | Def of  clinical response | Def of  clinical remission | Def of  endoscopic remission | Clinical  response | Clinical  remission | Endoscopic remission |
| Chen et al.^39^ | 12 weeks | Mayo score reduce by ≥ 3 | Total Mayo subscore ≤ 1 for rectal bleeding  plus stool frequency | Mayo endoscopic subscore ≤ 1 | 34/44 (77.3%) | 30/44 (68.2%) | 0 |
| Li et al.^11^ | Every 3 months after the initial FMT | Partial Mayo score reduce by ≥ 3 and ≥ 20% | Partial Mayo score ≤ 1 | - | 7/22 (31.8%, sustained response at 4 months) | 7/22 (31.8%) | - |
| Ohmiya et al.^36^ | 8 weeks | UC: Mayo score reduce by ≥ 3；  CD: CDAI reduce by ≥ 70 | UC: Mayo score < 3 and endoscopic Mayo score = 0;  CD: CDAI < 150 | - | UC 9/28 (32%), CD 3/4 (75%) | UC 4/28 (14%), CD 1/4 (25%) | - |
| Schierová et al.^54^ | 12 weeks | Mayo score reduce by ≥ 2 | Mayo score ≤ 2, with no subscore > 1 | Mayo endoscopic  score = 0 | 5/8 (62.5%) | 3/8 (37.5%) | 1/8 (12.5%) |
| Zhang et al.^19^ | 3 months | UC: Partial Mayo score reduce by ≥3 and ≥30%;  CD: HBI reduce by ≥3 | - | - | UC: NR  CD: 22/41 (53.7%) | - | - |
| Rossen et al.^29^ | 12 weeks | SCCAI reduce by ≥ 1.5 | SCCAI ≤ 2 | Endoscopic response: Mayo endoscopic  score of the sigmoid and rectum reduce ≥ 1 | 11/23 (47.8%) vs 13/25 (52.0%)^*^ | 7/23 (30.4%) vs 8/25 (32.0%), *P* = 1.0 | 8/23 (34.7%) vs 9/25 (36.0%), *P* = 1.0 |
| Table S4. Continued |  |  |  |  |  |  |  |
| Author | Follow-up | Def of  clinical response | Def of  clinical remission | Def of  endoscopic remission | Clinical  response | Clinical  remission | Endoscopic remission |
| Fuentes et al.^38^ | Median: responders 19.5 months, non-responders with sustained remission 17 months | NR | SCCAI ≤ 2 and  Mayo endoscopic  score of the sigmoid and rectum reduce by ≥ 1 | NR | NR | NR | NR |
| Paramsothy et al. (2017^37^, 2019^49^) | 8 weeks | Mayo score reduce by ≥ 3 or ≥ 50% reduction from  baseline in combined rectal bleeding plus stool frequency  Mayo subscore | Combined Mayo subscores ≤ 1 for  rectal bleeding + stool frequency | Mayo endoscopic subscore = 0 | 22/41 (54%) vs 9/40 (23%), *P* = 0.004 | 18/41 (44%) vs 8/40 (20%), *P* = 0.021 | 5/41 (12%) vs 3/40 (8%), *P* = 0.48 |
| Costello et al.^42^ | 8 weeks | Mayo score reduce by ≥ 3 | SCCAI ≤ 2 | Mayo endoscopic subscore ＜1 | 21/38 (55%) vs 8/35 (23%), OR 4.3 (*P* = 0.007) | 18/38 (47%) vs 6/35 (17%), OR 4.5 (*P* = 0.01) | 4/38 (11%) vs 0/35 (0%), OR 4.3 (*P* = 0.12) |
| Sokol et al.^40^  Kong et al.^41^ | 24 weeks | A clinical flare was defined as a CDAI > 220  points, by a CDAI between 150 and 220 with an increase  > 70 compared with baseline, or by the need for surgery  or to start a new medical treatment for CD | | | NR | 7/8 (87.5%) vs 4/9 (44.4%) | NR |

CAI, Lichtiger’s Clinical Activity Index ; CD, Crohn’s disease; CDAI, Crohn’s Disease Activity Index; FCP, F-calprotectin; FMT, fecal microbiota transplantation; HBI, Harvey–Bradshaw Index; NR, not recorded; PCDAI, Paediatric Crohn’s Disease Activity Index; PUCAI, Paediatric Ulcerative Colitis Activity Index; SCCAI, Simple Clinical Colitis Activity Index; SES-CD: Simplified Endoscopic Activity Score - Crohn’s disease; UC, ulcerative colitis. ^*^: FMT group vs Control group.

Table S5. Methods for sample collection, detection and analysis

| Author | Timepoint of  sample collection | Type of specimen | Microbiota assessment method (target region) | Sequencing  platform | Data analysis  tool | Reference  database |
| --- | --- | --- | --- | --- | --- | --- |
| Kao et al.^14^ | 1 day before  FMT, 7 days, 12 days, and 4 weeks after FMT | Stool | Multi-tag pyrosequencing  (16S rRNA) | GS-Junior pyrosequencer (Roche) | NR | RDP |
| Shimizu et al.^15^ | 0, 3, 16, 32 weeks | Stool | NR | Illumina HiSeq 2000 | MePIC, MEGAN viewer | NR |
| Quagliariello et al.^16^ | 0, 4, 8, 12, 16 weeks | Stool | 16S rRNA sequencing (V3-V4 region) | Illumina MiSeq DX | QIIME | Greengenes |
| Angelberger et al.^17^ | 0, 1, 2, 4, 8, 12 weeks | Stool | Pyrosequencing  (16S rRNA V6-V9 region) | GS FLX (Roche) | Mothur, QIIME | RDP |
| Suskind et al.^18^ | 0, 2, 6, 12 weeks | Stool | Metagenomic sequencing | Illumina HiSeq  2000 or MiSeq | MetaPhlAn | NR |
| Vaughn et al.^25^ | 0, 4, 8 weeks | Stool | Whole-genome shotgun sequencing | Illumina HiSeq 2500 | MetaPhlAn 2.0, HUMAnN2 | NR |
| Vermeire et al.^30^ | 0, 1, 2, 4, 6, 8, 24 weeks | Stool | 16S rRNA amplicon sequencing (V3-V5 region) | 454 GS-FLX sequencer | Mothur | RDP |
| Jacob et al.^32^ | 0, 2, 4 weeks | Stool | 16S rRNA amplicon sequencing (V4 region) | Illumina MiSeq | Usearch | Silva |
| Ishikawa et al.^21^ | Baseline, 2 weeks after antibiotics therapy, 4 weeks after FMT | Stool | 16S rRNA amplicon sequencing (entire region) | Illumina MiSeq | MEGAN | NR |
| Table S5. Continued | | | | | | |
| Author | Timepoint of  sample collection | Type of specimen | Microbiota assessment method (target region) | Sequencing  platform | Data analysis  tool | Reference  database |
| Nishida et al.^33^ | 8 weeks | Stool | PCR and T-RFLP analysis (16S rRNA) | ABI PRISM 3100 genetic analyzer | GeneScan 3.1 | Microbiota profiler (InfoCom T-RFLP Database & Analysis  Software) |
| Goyal et al.^23^ | 1 week, 1 month, 6 months | Stool | 16S rRNA amplicon sequencing (V4 region) | Illumina MiSeq | QIIME | NR |
| Karakan et al.^31^ | NR | Stool | 16S rRNA amplicon sequencing | NR | NR | NR |
| Kump et al.^22^ | 1, 3, 14, 28, 42, 56, 90 days | Stool | 16S rRNA amplicon sequencing (V4 region) | Illumina MiSeq | Mothur | Silva, Greengenes |
| Nusbaum et al.^26^ | 0, 4 weeks | Stool | 16S rRNA amplicon sequencing (V4 region); shotgun metagenomic sequencing | Illumina MiSeq; Illumina NextSeq500 | QIIME; CAFE | Greengenes; Reference database from NCBI |
| Cold et al.^24^ | 0, 2, 4, 8, 12, 16, 24 weeks | Stool | 16S rRNA amplicon sequencing (V3-V4 region) | Illumina MiSeq | Usearch | RDP |
| Fan et al.^27^ | NR | Stool | 16S rRNA amplicon sequencing | NR | NR | NR |
| Gogokhia et al.^35^ | NR | Stool | 16S rRNA amplicon sequencing | NR | NR | NR |
| Gutin et al.^34^ | Baseline, 6 +/- 2 weeks post-FMT | Stool | 16S rRNA amplicon sequencing (V4 region) | Illumina NextSeq 500 | QIIME, Usearch | Greengenes |
| Table S5. Continued | | | | | | |
| Author | Timepoint of  sample collection | Type of specimen | Microbiota assessment method (target region) | Sequencing  platform | Data analysis  tool | Reference  database |
| Chen et al.^39^ | Baseline, 4, 12 weeks | Stool | 16S rRNA amplicon sequencing (V4, V3-V4, V4-V5 region) | Ion S5™ XL system (Thermo Fisher) | QIIME | NR |
| Li et al.^11^ | Baseline, 5 days | Stool | 16S rRNA amplicon sequencing (V4-V5 region) | Illumina MiSeq | Mothur | NR |
| Ohmiya et al.^36^ | NR | Stool | 16S rRNA amplicon sequencing (V1-V2 region) | Illumina MiSeq | QIIME2 | NR |
| Schierová et al.^54^ | 0, 2, 4, 6, 12 weeks | Stool | 16S rRNA amplicon sequencing (V4-V5 region) | Ion Torrent PGM platform (Thermo Fisher) | QIIME2, DADA2 | Greengenes |
| Zhang et al.^19^ | Baseline, 3 months | Stool | 16S rRNA amplicon sequencing (V4-V5 region) | Illumina MiSeq | Mothur | NR |
| Rossen et al.^29^ | 0, 6, 12 weeks | Stool | HITChip (16S rRNA) | HITChip | NR | NR |
| Fuentes et al.^38^ | 0, 12 weeks, ≥ 1 year | Stool, rectum biopsy | HITChip (16S rRNA) | HITChip | NR | Greengenes |
| Paramsothy et al. 2017^37^ | Baseline, 4 and 8 weeks of masked, 8 weeks after completion of masked | Stool | 16S rRNA amplicon sequencing | Illumina MiSeq | Mothur | RDP, Silva |
|  |  |  |  |  |  |  |
| Table S5. Continued | | | | | | |
| Author | Timepoint of  sample collection | Type of specimen | Microbiota assessment method (target region) | Sequencing  platform | Data analysis  tool | Reference  database |
| Paramsothy et al. 2019^49^ | Baseline, 4 and 8 weeks of masked, 8 weeks after completion of masked | Stool, colonic biopsy | 16S rRNA amplicon sequencing, shotgun metagenomics | Illumina MiSeq, HiSeq 2500 | Mothur; MetaPhlAn2, HUMAnN2 | RDP, Silva |
| Costello et al.^42^ | 0, 4, 8, 52 weeks | Stool | 16S rRNA amplicon sequencing (V4-V5 region) | Illumina HiSeq 2500 | Usearch | RDP |
| Sokol et al.^40^ | -2, 0, 2, 6, 10, 14, 18, 24 weeks | Stool | 16S rRNA amplicon sequencing (V3-V4 region) | Illumina MiSeq | QIIME | Silva |
| Kong et al.^41^ | -2, 0, 2, 6, 10, 14, 18, 24 weeks | Stool | Metagenomic shotgun sequencing | Illumina NovaSeq 6000 | MetaPhlAn, HUMAnN2, StrainPhlAn | NR |

FMT, fecal microbiota transplantation; HITChip, Human Intestinal Tract Chip; PCR, Polymerase chain reaction; T-RFLP, terminal restriction fragment length polymorphism; RDP, Ribosomal Database Project; rRNA, ribosomal RNA.
